# Challenges of Treating ADHD with Comorbid Substance Use Disorder: Considerations for the Clinician

**DOI:** 10.3390/jcm12093096

**Published:** 2023-04-24

**Authors:** Margherita Barbuti, Marco Maiello, Vincenza Spera, Alessandro Pallucchini, Giulio E. Brancati, Angelo G. I. Maremmani, Giulio Perugi, Icro Maremmani

**Affiliations:** 1 2nd Psychiatric Unit, Department of Clinical and Experimental Medicine, Santa Chiara University Hospital, University of Pisa, 56100 Pisa, Italy; margherita.barbuti@hotmail.com (M.B.); marcomaiello@aol.com (M.M.); e.spera@hotmail.it (V.S.); pallucchini.a@gmail.com (A.P.); giuliobrancati@gmail.com (G.E.B.); giulio.perugi@med.unipi.it (G.P.); 2 Section of Psychiatry, Department of Psychiatry and Addictions, North-Western Tuscany Local Health Unit, Tuscany NHS, Versilia Zone, Via Aurelia 335, 55041 Lido di Camaiore, Italy; angelo.maremmani@uslnordovest.toscana.it

**Keywords:** attention deficit/hyperactivity disorder, substance use disorder, stimulants

## Abstract

Adults with attention deficit/hyperactivity disorder (ADHD) often present psychiatric comorbidities and, in particular, substance use disorder (SUD). ADHD-SUD comorbidity is characterized by greater severity of both disorders, earlier age of onset, higher likelihood of polydrug-abuse and suicidal behaviors, more hospitalizations, and lower treatment adherence. At the present stage, research focused on the pharmacological management of ADHD with comorbid SUD in both adolescents and adults is still lacking. Furthermore, while the short-term effects of stimulants are well studied, less is known about the chronic effects of these drugs on dopamine signaling. Current available evidence is consistent in reporting that high doses of stimulant medications in ADHD-SUD subjects have a mild to moderate efficacy on ADHD symptoms. Some data suggest that pharmacological treatment with stimulants may be beneficial for both ADHD symptoms and comorbid cocaine or amphetamine use. However, in the long run, stimulant medications may have a potential risk for misuse. For the absence of potential misuse, atomoxetine is often recommended for ADHD with comorbid cocaine or amphetamine use disorder. However, its efficacy in reducing addictive behavior is not demonstrated. In subjects with other subtypes of SUD, both atomoxetine and stimulant drugs seem to have scarce impact on addictive behavior, despite the improvement in ADHD symptomatology. In this population, ADHD treatment should be combined with SUD-specific strategies.

## 1. Introduction

Attention deficit/hyperactivity disorder (ADHD) is characterized by symptoms of inattention, distractibility, disorganization, hyperactivity, and both behavioral and emotional impulsivity. It is the most common neurodevelopmental disorder diagnosed in childhood and persists into adolescence and adulthood in about 60–70% of cases [1,2]. The worldwide prevalence of ADHD among children and adolescents is estimated between 5% and 7% [3], while, in adulthood, it ranges between 2% and 6% [4].

In children, the male-to-female ratio ranges from 2:1 to 10:1 and is higher in clinical samples than in the general population. With increasing age, this prevalence discrepancy between males and females diminishes [5]. Presumably, the externalizing clinical manifestations and behavioral disturbances, prominent in male children, lead more easily to diagnosing ADHD in this population. On the other hand, the clinical presentation of females more often leads to a missed diagnosis because of their tendency to present internalizing manifestations, such as mood and anxiety disorders, and their better social adjustment [6].

In adults, hyperactivity usually improves, while inattention, impulsivity, and emotional dysregulation persist or even worsen, resulting in social, interpersonal, and work impairment. In addition, most adults with ADHD show numerous psychiatric comorbidities, such as alcohol/substance use, mood, anxiety, eating, and impulse control disorders, which complicate the clinical picture and therapeutic management and are associated with poor treatment outcomes [7].

In particular, substance use disorder (SUD) comorbidity in ADHD subjects is very common, and nearly a quarter of young adults with SUD meet the diagnostic criteria for ADHD [8,9]. In general, in individuals with ADHD, SUD is more severe with earlier age of onset, higher likelihood of polydrug-abuse and suicidal behaviours, a higher rate of relapse, more hospitalizations, and lower treatment adherence [10,11]. All subtypes of ADHD are associated with SUD, although the combined type is more strongly related with externalizing disorders [12]. Therefore, screening for ADHD symptomatology in all patients seeking treatment for a substance use disorder is necessary, although not always obvious. In fact, some key features of ADHD, such as attention difficulties, hyperactivity, and impulsivity, are also part of SUD psychopathology, thus complicating differential diagnosis [13].

From a neurodevelopmental perspective, ADHD symptoms starts in childhood, suggesting their role as risk factors for early onset of substance use and the development of SUD. Childhood ADHD has been associated with a small to moderate increased risk of SUD in adolescence or early adulthood (with odds ratios ranging from 1.34 to 3.48 for different types of SUD) [14]. According to some studies, treatment with stimulants for ADHD during childhood may reduce the risk of developing SUD [15,16], representing a potential protective factor.

The dysfunctions of the brain’s inhibitory and reward systems, resulting in impulsivity and inability to delay gratification, that characterize ADHD are the main developmental risk factors for early substance use, misuse, and SUD in adolescence and early adulthood [17,18]. The dopaminergic transmission is involved in both ADHD and SUD pathophysiology [19]. Dopamine (DA) presides the neural circuits that control impulsivity and reward and is responsible for delayed gratification. Processes of “now” versus “later” require a fine tuning of DA release in top-down and bottom-up brain circuits. A steady release in the striatum and the prefrontal cortex, as in the tonic DA firing, mediates “later” processes, whereas sharp, fast bursts of DA drive “now” processes [20]. In ADHD individuals who have impaired dopaminergic transmission [21], substances of abuse increase the release of DA, reducing inattentive symptoms and inner restlessness.

The substances most commonly used by ADHD subjects are alcohol and/or nicotine, cannabinoids, stimulants (amphetamines and cocaine) and opiates [22,23]. The presence of negative emotionality in ADHD individuals seems more frequently associated with alcohol use, while impulsivity and conduct disorders are more common in subjects with stimulants and cannabis use [24].

## 2. Pharmacologic Treatment Strategies in ADHD Subjects with Comorbid SUD

Currently approved pharmacological agents for youths and adults with ADHD act by increasing DA and norepinephrine (NE) transmission and include stimulant (for example, methylphenidate and amphetamines) and non-stimulant medications (for example, atomoxetine, guanfacine, clonidine, and bupropion) [25]. All guidelines agree that stimulants, especially in ER formulations, are first-choice treatments for ADHD in both children and adults [26,27]. When stimulant medications are ineffective, poorly tolerated, or contraindicated, non-stimulant medications are considered. Overall, all pharmacological compounds approved for short-term treatment of ADHD have proved to be effective in improving clinical symptoms and executive functions [26] both in children and adults.

In some cases, psychiatric comorbidities (for example, a severe psychotic mood episode, severe addictive disorders, etc.) constitute a therapeutic priority. However, the coexisting symptoms of ADHD should always be considered during treatment management because they can strongly influence the clinical picture and the response to pharmacological treatment [28].

To date, there is a paucity of research focused specifically on the pharmacological management of ADHD in comorbidity with SUD in both adolescents and adults, and the few recommendations are largely based on practice or short-term clinical trials [23]. In adults, the use of stimulants in comorbid ADHD and SUD has always been challenging because of their addictive properties, which carry a greater risk of misuse, especially in individuals with a history of stimulant/cocaine abuse [29]. Concerning treatment management, in clinical practice, an important distinction is between ADHD subjects with and without stimulant/cocaine addiction.

## 3. ADHD with Comorbid Stimulant/Cocaine Use Disorder

The current literature shows that ADHD in cocaine consumers has been identified in a range between 15.1% and 25% of the cases [30,31,32]. Combined type ADHD is the most common in adults with cocaine use disorder (CUD) (72.8%) [33], while inattentive type ADHD individuals have the lowest risk of developing a CUD [34].

Imaging and post-mortem studies on chronic cocaine abusers showed an increase in the striatum concentration of dopamine active transporter (DAT) with respect to controls due to the chronic extracellular DA increase after multiple intoxications [35]. Similarly, a positron emission tomography (PET) study conducted on 18 ADHD adults before and after 12 months of methylphenidate (MPH) treatment revealed an up-regulation of DAT in the striatum of ADHD subjects compared to controls [36]. The neuro-adaptation characterized by the increase of DAT concentration due to the chronic cocaine action on DA and occupancy of the transporters could explain the reduced effectiveness of stimulants in these individuals [37].

Long-term treatment with stimulants at high dosages may be effective in controlling ADHD symptomatology in individuals with comorbid cocaine use disorder (CUD) [38,39,40]. In particular, the dose of stimulant drugs (MPH or amphetamines) should be about 40% higher in subjects with ADHD-CUD than in those with ADHD alone [41,42]. Several studies have shown greater efficacy of long-acting MPH at high doses (40–80 mg/day) in controlling ADHD symptomatology and comorbid CUD [29,43]. Other studies have pointed to a therapeutic benefit of 60–80 mg long-acting mixed amphetamine salts combined with cognitive behavioral therapy in reducing both ADHD symptoms and cocaine use [44,45,46]. Clinical trials comparing the results of human laboratory experiments are also consistent in supporting the efficacy of MPH (above 60 mg/day) for the treatment of CUD in subjects with ADHD [47,48]. However, negative results regarding the effectiveness of ADHD medications in reducing cocaine use in ADHD subjects have been reported [47,49].

While stimulant medications have shown some efficacy in prolonging cocaine withdrawal, regardless of comorbidity with ADHD [50], they appear to have little impact on amphetamine abuse or dependence [42].

In a recent meta-analysis of randomized-controlled studies, indeed, the benefits demonstrated by prescription stimulants in patients with stimulant use disorder were mainly driven by studies on CUD [51]. Although few trials were available for amphetamine-type stimulant use disorder (ATSUD), none of them reported positive effects of prescription stimulants compared to placebo on amphetamine abstinence [42,51]. Nevertheless, some advantages of MPH may be observed in ADHD-ATSUD patients when extremely high doses are used [52]. As far as we know, only two double-blind trials have been published on MPH treatment of comorbid ADHD-ATSUD by the same group [52,53]. In the first pilot study, no differences between treatment groups were observed for ADHD symptom improvements, urine toxicology, and self-reported amphetamine use, craving, or retention in treatment in 24 adult outpatients randomized to receive 72 mg of extended-release MPH or placebo for 12 weeks [53]. Conversely, the second randomized-controlled trial showed that 24-week high-dose MPH treatment (mean dose = 168.6 ± 26.4 mg) was associated with greater improvements in ADHD symptoms, with a higher proportion of drug-negative urines and with better retention in treatment in 54 abstinent men released from medium security prisons [52]. While needing replication in different settings, these findings emphasize the need for a wide range of doses of MPH to achieve clinical response in patients with ADHD-ATSUD comorbidity. Given the paucity of studies, ATSUD comorbidity represents an area of unmet needs in the treatment of ADHD patients with SUD.

Another problem in the treatment of adults with ADHD-CUD/ATSUD is the potential misuse of stimulants, which, however, appears to be less common with MPH than with amphetamines and seems to diminish, at least in part, with long-acting formulations [29]. Atomoxetine is often recommended for ADHD with comorbid CUD; however, its efficacy in reducing addictive behavior has yet to be demonstrated [54]. Given its low abuse potential, bupropion (250–400 mg/day) may have a role in treating ADHD individuals with comorbid CUD and depression [55,56]. Finally, stimulant abuse may significantly contribute to treatment non-adherence in patients with ADHD. Particularly, stimulant use disorder has been found to predict MPH discontinuation in patients diagnosed with comorbid ADHD-SUD from the Swedish national registers [57].

## 4. ADHD with Comorbid Alcohol Use Disorder

The literature regarding the relationship between ADHD and SUD is mainly imprinted on cannabis or stimulant drugs [29,58], leaving a gap regarding comorbidity with alcohol use disorder (AUD). A lifetime prevalence of any AUD up to 43% [59] and a 3 to 11% prevalence of moderate to severe AUD has been reported in ADHD subjects [59,60], with higher rates in adults than in adolescents [60].

ADHD is considered a risk factor for developing AUD, with an odds ratio of 1.5 [61]. Presumably, there is an association between impulsivity, which is a nuclear symptom of ADHD, and younger age at onset of alcohol consumption, tendency to binge drink, and higher risk of relapse in abstinent AUD subjects [62]. In addition, attentional deficits were correlated with the amount of alcohol consumption in ADHD patients compared with controls [63]. The risk of AUD in ADHD individuals increases when comorbidities such as bipolar, conduct, antisocial personality, or eating disorders are present [64,65]. As expected, ADHD-AUD is associated with worse treatment outcome and a high number of comorbidities [66].

A common pathogenic pathway between ADHD and AUD is thought to be a reduced dopaminergic activity, with AUD subjects presenting a decreased release of DA in the limbic system, and a reduced availability of dopaminergic receptors [67], although both disorders are characterized by the involvement of other neurotransmitters [68].

On the matter of pharmacological treatment, one study analyzed the effects of atomoxetine on ADHD symptoms and alcohol use in a sample of currently abstinent ADHD/AUD individuals. Atomoxetine was effective in reducing ADHD symptomatology, but it did not have an impact on the relapse into heavy drinking [69].

## 5. ADHD with Comorbid Cannabis Use Disorder

Cannabis is the most commonly used illicit drug of abuse, the third if we consider the licit drugs (alcohol and tobacco). Considering the global population aged 15 to 64 years old, almost 4% used cannabis at least 1 time during 2019 [70], with an increased use over the past 10 years of almost 18%. Cannabis use disorder has been associated with several psychiatric disorders, in particular, ADHD. Adolescents with cannabis use disorder, cannabis abuse, and cannabis problem use received a diagnosis of ADHD in 33.2%, 34.6%, and 38.1% of the cases, respectively [71].

The intersection and often overlapping effects of cannabis and ADHD can be challenging for the clinician. Tetrahydrocannabinol (THC) binds to cannabinoid receptors in the brain, causing feelings of well-being, euphoria, and anxiolysis, but also altering systems involved in memory, concentration, and time perception [72]. Indeed, chronic cannabis consumption alters executive functions in a similar way that ADHD does [73], with impaired sustained attention and working memory [74], and possibly alters brain circuitry accountable for response inhibition [75], impulsivity [76], cognitive control [77], reward processing [78], circadian rhythms [79], and motivation [80].

Neurotransmitters such as DA, serotonin, norepinephrine, gamma-aminobutyric acid (GABA), acetylcholine, histamine, and opioid peptides are also influenced by cannabis [81]. In particular, THC stimulates the release of DA in the striatum and substantia nigra, increasing the salience of otherwise neutral stimuli [82]. Chronically, THC causes the depletion of the dopaminergic system, which is responsible for the onset of amotivational syndrome and executive function deficits [83]. Thus, cannabis use onset in ADHD subjects is led by reward craving, the desire for pleasurable and relaxing effects [84], but ultimately leads to worsening of ADHD symptoms [85]. In addition, use of cannabis during treatment makes it difficult to evaluate the benefits of ADHD medications on the symptoms of hyperactivity and inattention.

In a recent systematic review on cannabis use in ADHD, the authors did not report evidence of an impact of cannabis on cognition, measured by neuropsychological task performance, among users with ADHD compared to nonusers [86]. On the other hand differences were seen in brain morphology (decreased thickness in right and precentral and postcentral gyri [87] and increased thickness in left nucleus accumbens and right superior frontal and postcentral gyri [88]) and DA transporter density (lower in ADHD/SUD subjects) [89].

As a general guideline, in order to treat ADHD individuals with comorbid cannabis use, it is important to reduce its consumption and possibly use melatonin in case of circadian rhythm disorders. The only clinical trial that evaluated ADHD treatment on ADHD symptoms and cannabis use found that atomoxetine, when compared to placebo, reduced ADHD symptoms, but it did not impact cannabis use, after 12 weeks of treatment [90].

## 6. ADHD with Comorbid Opioids Use Disorder

Opioids are the least common drug of abuse among ADHD subjects, and the ADHD/opioids comorbidity is thoroughly understudied. The comorbid diagnosis of ADHD among opioid use disorder (OUD) subjects seeking treatment is estimated to be around 20–25% [91,92] and leads to a reduced compliance to the methadone treatment [93] and higher rates of dropout [94]. ADHD-OUD subjects may enter methadone maintenance treatment more frequently than non-ADHD subjects [95] due to a greater tendency for risky behaviors and injuries, including those from motor vehicle accidents [96].

ADHD subjects who develop OUD are characterized by more severe addiction and comorbid psychopathology, only in part explained by the coexistence of a conduct disorder [97]. Effective treatments for ADHD/OUD subjects have been limited and seem to be influenced by a scarce compliance to ADHD medication, with 50% of individuals discontinuing treatment within the first two years due to side effects, illicit drug use, and low psychosocial functioning [98].

A study compared the effects of MPH, bupropion, and placebo on a cohort of methadone-maintained subjects [99]. The results did not show any impact of MPH or bupropion on drug use, while improvement in ADHD symptomatology has been observed in all three groups (MPH, bupropion, and placebo). On the other hand, encouraging results were obtained from a small naturalistic study [100] conducted in a Swedish substance use disorder treatment facility. Treatment with opioid substitutes (methadone or buprenorphine) and central stimulants (long-acting MPH or modafinil) in ADHD subjects with OUD led to a reduction in opioid use and ADHD symptoms. Similarly, in another naturalistic study, 20 ADHD subjects on opioid maintenance treatment received long-acting stimulant medication and reported improvement in ADHD symptoms during follow-up, although some residual symptoms remained, suggesting persistent functional impairment [98]. It should be noted that in this study, 50% of the sample also had a stimulant use disorder. This finding is a limitation for the study’s conclusions but reflects real-world clinical practice, in which clinicians are faced with subjects who often engage in polydrug abuse.

In conclusion, there is no clear evidence on when to start ADHD-specific treatments in subjects with OUD. Most studies seem to indicate that co-medication with ADHD treatment should be started after stabilization of opioid maintenance treatment.

## 7. Long-Term Outcomes of Drug Treatment in ADHD Subjects with Comorbid SUD

While a large amount of studies confirmed the beneficial effects of psychostimulants in the acute treatment of ADHD both in childhood and adulthood, fewer works address the chronic and long-term effects of these treatments on brain DA transmission, ADHD symptomatology, and functional outcomes. A systematic review of follow-up studies confirmed the greater efficacy of ADHD medication (stimulants and atomoxetine) over placebo in 5 randomized clinical trials and 11 open label extension studies of at least 24 weeks, with some naturalistic studies showing a possible role of ADHD medications in preventing depression and SUD in adulthood [101]. Regarding the risk of SUD in adult ADHD subjects, a first metanalysis concluded that stimulant medication use during childhood is a protective factor towards the development of SUD, probably due to the improvement of ADHD symptomatology [102]. However, a more recent metanalysis obtained different results, suggesting that treatment with stimulant medications neither protects nor increases the risk of SUD [103].

While the acute effects of stimulants are well studied, less is known about the chronic effects of these medications on DA signaling. In fact, long-term treatment with stimulants leads to neuroadaptive changes that could explain the loss of their therapeutic efficacy observed in ADHD individuals [37]. This effect seems to be particularly pronounced in ADHD subjects with comorbid SUD, in whom stimulants are prescribed at higher dosages than in ADHD subjects without SUD [104].

A single-photon emission computed tomography (SPECT) study analyzed DAT availability and the effect of MPH on DAT in ADHD subjects with and without cocaine dependence (CUD). At the baseline evaluation, ADHD-CUD subjects, who were abstinent for about 1.5 years, had a 25% lower DAT concentration in the striatum and a lower DAT occupancy after 2 weeks of MPH treatment compared to ADHD subjects without SUD. In this study, MPH response was less pronounced in the ADHD with CUD population than in those without. However, the difference between the two groups was not statistically significant. These findings are in line with other controlled studies showing small effects of MPH treatment in ADHD individuals with CUD [37].

Overall, scientific data on the long-term effects and, consequently, the recommended duration and doses of prescribed stimulant treatment in adults with ADHD are still unconclusive, with few high-quality studies. From a clinical point of view, the use of long-lasting stimulant treatments for ADHD symptoms, even at high doses in ADHD-SUD individuals, seems to have a limited long-term efficacy and still a potential risk of misuse and addiction.

## 8. Conclusions

Although, in the clinical setting, SUD is one of the most common comorbidities in adult ADHD subjects, data on the impact of SUD on treatment response of ADHD symptoms with stimulant medications or atomoxetine are still scarce. In particular, none of these drugs have been tested in long-term clinical trials, not allowing their long-term effectiveness to be clarified. Furthermore, many ADHD subjects that psychiatrists deal with in routinary clinical practice, who often have several psychiatric comorbidities and reduced adherence to treatment, are usually excluded from clinical trials. In light of these important gaps, the International Collaboration on ADHD and Substance Abuse (ICASA) foundation designed and conducted the International Naturalistic Cohort Study of ADHD and SUD (INCAS) [23]. This prospective, international cohort study aims to comprehensively investigate the treatment modalities provided to ADHD subjects with comorbid SUD, to describe the clinical course of these individuals, and to identify predictors of treatment outcomes. At present, only the protocol and baseline clinical characteristics of the sample have been published.

According to some authors [105], interventions on substance abuse are the primary focus in the management of individuals with ADHD-SUD comorbidity. In light of this view, specific treatment for ADHD-related symptomatology should be considered only after a period of abstinence from substances has been achieved. However, untreated ADHD is a known risk factor for relapse to substance use and has been shown to negatively impact treatment compliance. Consequently, substance abstinence may be an unrealistic goal to achieve in the case of individuals with marked ADHD symptomatology. It is therefore critical to engage these individuals in integrated treatment programs in which ADHD treatment emerges as a primary goal along with SUD-specific strategies.

Indeed, the reported improvement of ADHD symptoms in subjects with comorbid SUD should not discourage ADHD treatment (see Box 1). The overall tolerability profile of stimulant and non-stimulant preparations suggests that these drugs are probably safe when used to treat patients with co-occurring SUD-ADHD [22]. In addition, in some individuals with predominantly inattentive ADHD and comorbid cocaine or amphetamine use, pharmacological treatments for ADHD may also have a positive impact on addictive behaviors. In subjects with other types of SUD, although improvement in ADHD symptoms has been reported with both stimulants and atomoxetine, these drugs seem to have scarce impact on addictive behavior.

Box 1 **Summary.**

- Attention deficit/hyperactivity disorder (ADHD) may represent a risk factor for early-onset substance use disorder (SUD).
- Treating ADHD symptoms during childhood or adolescence with stimulant medications may reduce the risk of developing SUDs in adulthood.
- Comorbidity between ADHD, especially the combined type, and SUD is very common and results in increased severity of both disorders.
- The substances most commonly used by ADHD individuals are stimulants (cocaine, amphetamines), cannabinoids, alcohol, and opiates.
- In subjects with comorbid cocaine or amphetamine use disorder, high-dose stimulant medications are needed to improve ADHD symptomatology.
- In individuals with predominantly inactive ADHD, stimulant medications may also have a positive impact on cocaine withdrawal. Less consistent data have emerged regarding the impact of these medications on amphetamine use disorder.
- In individuals with alcohol, cannabis, or opiate use disorder, pharmacological therapy for ADHD appears to have little impact on addictive behaviors, despite improvement in ADHD symptomatology.
- Despite the paucity of studies on pharmacological management of ADHD-SUD comorbidity, substance abuse should not discourage ADHD treatment.
- In individuals with ADHD-SUD comorbidity, especially those with a history of amphetamine/cocaine abuse, attention should be paid to the risk of stimulant misuse.
- Methylphenidate has a lower risk of abuse than amphetamines, especially in extended-release formulations.
- Atomoxetine is often recommended for individuals with ADHD in comorbidity with cocaine use disorder, but its effectiveness in reducing addictive behavior has yet to be proven.
- Given its low abuse potential, bupropion may have a role in treating ADHD individuals with comorbid cocaine abuse and depression.
- Although the beneficial effects of psychostimulants in the acute treatment of ADHD are widely confirmed, stimulants may lose their therapeutic efficacy in the long term, especially in comorbid SUD subjects.

Obviously, attention should be paid to the risk of abuse of stimulant medications in ADHD-SUD individuals, especially if they use cocaine or amphetamines. Close monitoring for signs of abuse, such as not attending appointments or requesting higher doses or more prescriptions of the drug, is essential after initiating treatment. In this population, extended-release MPH formulations are recommended. For those with active SUD and/or high risk of abuse, non-stimulants, such as atomoxetine and bupropion, should be considered [25].

Alongside pharmacological treatment, psychotherapeutic interventions play a role in the management of ADHD-SUD subjects. Indeed, psychoeducation of patients and family members to enhance knowledge about the condition, natural history, and prognosis are critical to improve recognition and treatment of the ADHD-SUD comorbidity [22]. In addition, structured and adapted psychotherapies, such as motivational enhancement, CBT, and/or contingency management can be helpful in the treatment of these individuals [106].

Finally, the wide range of other psychiatric conditions often associated with ADHD in adults suggest to adopt an individualized therapeutic plan and not only an ADHD targeted treatment.

In the future, larger randomized clinical trials and naturalistic studies with longer follow-ups in adult subjects with ADHD and comorbid SUD are needed to better define the long-term efficacy and safety of ADHD medications.

## Data Availability

Not applicable.
